# Patient Participation in Surgical Treatment Decision Making from the Patients' Perspective: Validation of an Instrument

**DOI:** 10.1155/2012/939675

**Published:** 2012-07-04

**Authors:** Liv-Helen Heggland, Torvald Øgaard, Aslaug Mikkelsen, Kjell Hausken

**Affiliations:** ^1^Surgical Division, Stavanger University Hospital, 4011 Stavanger, Norway; ^2^Norwegian School of Hotel Management, University of Stavanger, 4036 Stavanger, Norway; ^3^Business School, University of Stavanger, 4036 Stavanger, Norway; ^4^Faculty of Social Sciences, University of Stavanger, 4036 Stavanger, Norway

## Abstract

The aim of this paper is to describe the development of a new, brief, easy-to-administer self-reported instrument designed to assess patient participation in decision making in surgical treatment. We describe item generation, psychometric testing, and validity of the instrument. The final scale consisted of four factors: information dissemination (5 items), formulation of options (4 items), integration of information (4 items), and control (3 items). The analysis demonstrated a reasonable level of construct validity and reliability. The instrument applies to patients in surgical wards and can be used to identify the health services that are being provided and the areas that could strengthen patient participation.

## 1. Introduction

In the relationship between healthcare professionals and patients, there is an imbalance in knowledge regarding disease, prognosis, and treatment. Patients' knowledge has been assumed to be weak. The knowledge, competence, and expertise of healthcare professionals have been assumed to be strong and with the capacity to define patients' best interests [1]. Based on their position and professional competence, the authority of healthcare professionals has regulated the degree to which patients are allowed to contribute to their treatment situation. The traditional view of the relationship between professionals and patients has recently been challenged from a different number of perspectives. Formally, regulations [2–5] and developing views on health services management, patients have increasingly been encouraged to be more active in their treatment and care. Furthermore, management-oriented leadership reforms have promoted the patient-as-partner view, maintaining that the patient is a participating producer of healthcare, and new social trends, including increased public access to information and knowledge via the internet. This has opened for some balancing of the information gap between professionals and patients: patients can use the internet to access information about disease progression, treatment alternatives, rights and choices, medical results, and unique qualities of hospitals or clinics. The emerging view is that healthcare professionals now meet patients as costumers who buy services and want choices, quality, and service [6]. From a customer perspective, the patient may demand a partnership with healthcare professionals. The customer may want information about the service being purchased and a guarantee that the service is of high quality. Patients as costumers purchase services and demand availability, quality, and service [6]. All of these trends imply that communication and dialogue between healthcare professionals and patients have become important.

However, recent research seems to indicate that the patient perspective is not given sufficient attention [7]. In their Danish study Freil and Knudsen [7] found that one out of five patients reports insufficient involvement in his or her course of treatment. They assert that there is a general lack of user representation and surveys in the processes that shape health services organization and cultural issues. Research on shared decision making is young, and new measurement instruments for decision making processes, outcomes and surrounding elements are constantly being developed [8, 9]. Few measurement instruments are widely used, and the extent of validation of scales differs. Most instruments are self-reported scales which were tested for factorial validity without investigation of convergent and discriminant aspects of validity, and few of them were compared with each other [9].

The objective of this paper is to develop and investigate the psychometric properties of a self-reported instrument that measures patient participation in decision making in surgical treatment from the patients' perspective. The data for the study were gathered from patients who have undergone surgical treatment. We investigated the scope of patient participation through an analysis of Norwegian surgical patients' and healthcare professionals' beliefs and practices related to patient participation in the decision making processes in surgical treatment (i.e., operations or surgical exploration).

## 2. Methods

### 2.1. Instrument Development

The instrument development consisted of four steps [10]. First, a literature review was conducted on concepts and models of patient participation in decision making. We reviewed the literature using the words patient participation, patient involvement, and user involvement in surgical treatment in the Academic Search Elite, CINAHL, MEDLINE, Cochrane, PsycINFO and ISI databases, and in Norwegian public documents on the subject of patient participation and user involvement. According to Cahill [11] an analysis of “patient participation” showed that the meaning of the concept is comparable to patient partnership, patient collaboration, and patient involvement. 

The literature review indicated that particularly important aspects of patient participation are empowerment [12], participation in treatment decision-making processes [13–15] and active versus passive patient roles in treatment [16–18]. These three general theoretical constructs were then included in an interview guide that focused on patients and healthcare professionals' respective views on patient participation. The interviews were structured around the following questions: “What does patient participation in surgical treatment imply?”, “What preconditions facilitate patient participation in surgical treatment?”, “What may hinder and promote patient participation in surgical treatment?”, and “What are the possibilities to strengthen patient participation in surgical treatment?”

Secondly, qualitative interviews were conducted with four physicians and seven nurses from six surgical wards and seven patients who had undergone surgical treatment. The interviews identified domain dimensions to measure patient participation in decision making for surgical treatment. Finally, a qualitative content analysis was performed. The interviews were conducted during two weeks in 2008. Applying purposive sampling [19–21], the eighteen participants were selected to ensure a broad representative sample, with experts of varying ages and sexes from medicine and nursing.  Table 1 presents the participants' characteristics. The head managers of six surgical wards selected the participants. The healthcare professionals' ages ranged from 26–62 years. The patients' ages ranged from 49–65 years. The healthcare professionals were questioned about their general perceptions about medical/surgical decision making. The two patients identified in Table 1 as “Back operation” and “Hip replacement” were interviewed two days before surgery (These two patients were moved from the orthopedic ward to rehabilitation at another institution after surgery and could not be tracked thereafter because of anonymity.) and the five remaining patients were interviewed 2–7 days after surgery. The aim of the interviews was to identify domain dimensions for measuring patient participation in decision making in surgical treatment. Applying qualitative interview criteria we concluded that 18 interviews were sufficient to achieve saturation.

Third, qualitative content analysis was conducted using the methodological logic suggested by Graneheim and Lundman [22]. Based on a frequency count, four theoretical categories were defined as important for measuring patient participation in decision making in surgical treatment: information dissemination, formulation of options, integration of information, and control. The qualitative analysis is described in detail elsewhere [23].

The first category, information dissemination, contains patients' opportunity to talk with answerable doctors and nurses upon admission. Nurses give the patients general information about the hospital stay, the anaesthetics, and the preparations before surgery, and the doctors give the patients information about surgical procedures, treatment options, and consequences. The second category, formulation of options, is the patient's possibilities to choose among options. The third category, integration of information, is about making the information understandable to the patients. Nurses distinguish between the nurses' and the doctors' information to the patients. Patients describe a lack of possibilities to talk with physicians and that nurses were unable to take responsibility about treatment decisions. Category four pertains to control which means rejection versus acceptance of paternalism and indicates patients' role in decision making in treatment.

The fourth step of the instrument development was to generate measurement (questionnaire) items. To ensure that the domain of the concept had been explored according to the four theoretical categories, we generated 12 items per category, resulting in a pool of 48 items. Following the recommendations from Churchill [24], validity and item generation included an evaluation of face and content validation of the items by an expert panel. The members of the expert panel consisted of four individuals with formal qualifications in health and social sciences. The panel gave feedback about the face validity of the items in terms of wording and clarity, and they assessed whether the items belonged together in the four theoretical categories. Their rating assessed apparent internal consistencies. Another basic requirement for content validity was the items' ability to capture the meaning of the four categories. The members were instructed to associate items with each of the four categories, to comment on unclear or overlapping items and to give comment about the items' and categories' ability to capture patient participation in decision making in surgical treatment. Duplication of statements, redundant items, and meaningless or confusing wording was avoided. This process resulted in 20 items that were further developed into questions in a questionnaire.

### 2.2. Empirical Testing of the Instrument

The empirical testing of the instrument was conducted by distributing a questionnaire and written information about the research to patients (*N* = 4,000). The 4,000 patients were selected randomly from nine surgical wards at a Norwegian university hospital. 1,048 patients answered the survey for a response rate of 26%. The characteristics of the sample are presented in Table 2. The patients had all undergone surgical treatment in the three months prior to receiving a questionnaire, from February to June 2009. The questionnaire was sent to their addresses from the hospital. The first 2,000 questionnaires were sent in April 2009, 1,000 questionnaires were sent in May 2009, and the last 1,000 were sent in June 2009. Most of the questionnaires were returned within one week of being sent. No additional incoming questionnaires were included for analysis after June 2009.

The hospital management consented to the participation, and the study was approved by the hospital research director, the head of the clinic at Stavanger University Hospital, the Norwegian Ethical Committee (Institutional Review Board) (no. 3.2007.1984) and the Norwegian Social Science Data Services (no. 17468). All potential respondents were provided with a full explanation of the study and were invited to participate. Prior to obtaining consent, potential respondents were reassured that their decision to participate was voluntary and that they were free to withdraw from participation at any time [2]. Full confidentiality was guaranteed. The completed questionnaires were returned anonymously with an included stamped and addressed envelope. All items on the questionnaire were structured with a Likert scale from 1 (strongly disagree) to 7 (completely agree) except for the background variables. Informed consent was implied by returning the questionnaire.

### 2.3. Plan for the Statistical Analysis

SPSS [25] was used for the statistical procedures. Empirical validation of the instrument followed the approach advocated for psychometric analysis by Churchill [24] and Pett et al. [26]. First the univariate distribution of items was assessed by calculating items' mean, standard deviation, skewness, and kurtosis [27]. Then, an initial assessment of convergent validity was done by a principal component factor analysis for each of the four theoretical dimensions of customer participation followed by reliability evaluation by calculations of Cronbach's alpha. To further investigate convergent and discriminant validity, all the 20 items were entered in a factor analysis [24, 26]. Finally, a further assessment of discriminant validity was done by calculating the correlations between the sumscores of each of the four dimensions.

The suitability of data for factor analysis was assessed by inspection of the correlation matrix, by computing the The Kaiser-Meyer Olkin value (KMO) [28] and by running Bartlett's Test of Sphericity [29]. KMO values of .60 or greater and a significant Bartlett's test (*P* < .05) for factor analysis were considered to be appropriate. For the factor analysis, at least three items per component were recommended, and for the correlation matrices the presence of coefficients greater than .30 was recommended [30].

Finally, the nomological validity of the measurement scale was assessed by evaluating the relationship between the four factors and the variables: “I had no influence on treatment” [31], and a Norwegian version of the Utrecht Coping List (UCL) [32]. The first variable focuses on subjective perceptions of fairness in treatment and justice in organizational settings and was expected to have a negative relationship to the “patient participation in decision-making in surgical treatment scale.” The UCL coping scale (9 items) relates to the belief that one is able to respond to, and control, external situations. Coping was assumed to be positively related to patient participation. These hypothesized relationships were investigated with a correlation analysis. The results of the analyses are reported below.

## 3. Results

### 3.1. Distribution of Items

Mean scores, standard deviation, skewness, and kurtosis values of 20 items are presented in Table 3. The skewness values ranged between −.11 to 1.64 and kurtosis values ranged between −.03 to 2.24, indicating normally distributed items [27]. No items were excluded because of poor distribution. Cronbach's alpha was used to assess factor reliability. As the Cronbach's alpha value of each factor was greater than .60, the internal consistency and reliability were considered to be adequate [33].

### 3.2. Factor Analyses

Convergent validity of the four factors was assessed by factor analysis. The first factor of each analysis captured a considerable amount of variance of each scale (50%–61%), while each of the items' lowest loadings on the first factor ranged from .62–.77, all indicating convergent validity.

Convergent and discriminant validity was further investigated by an exploratory factor analysis of all 20 items. The Kaiser-Meyer-Olkin (KMO) measure of sampling adequacy increased to .90, indicating a factorable correlation matrix [28] and Bartlett's test of Spherity reached statistical significance (*P* = .005) for the 20 items [29]. 

The exploratory analysis found four factors with eigenvalues greater than 1. (6.30–1.17, see Table 4). These empirical factors correspond closely to the theoretical dimensions (1) information dissemination, (2) formulation of options, (3) integration of information, and (4) control [23], but some cross-loadings and low factor loadings indicated that there was still room for improvement.

After deleting four items, all items loaded over .30 on one factor and had low loadings on other factors [30]. The result of a final analysis of the four-factor scale with 16 items is shown in Table 5.

Cronbach's alpha was used to assess factor reliability and ranged from .63 to .80. The two lowest alpha scores were estimated for the factors formulation of options (.65) and control (.68). The low alpha scores for these factors may be considered in relation to the factors being measured using four and three items, respectively; the number of items is a very important statistical parameter that influences the estimation of Cronbach's alpha [30, p. 230].

Nomological validity was assessed with a bivariate correlations analysis between the four dimensions of patient participation and the variable “I had no influence on surgical treatment” [31] and the UCL coping scale [32]. The correlation between first variable and the four factors varied between −.10 and −.21 (*P* < .001). The UCL coping scale correlated .53, .29, .66, .39 (*P* < .001) with the four participation dimensions, respectively (Table 6).

## 4. Discussion

The aim of this study was to develop, empirically test, and validate an instrument specifically designed to understand patient participation in decision making in surgical treatment. Empirical validation of the instrument followed the approach advocated for psychometric analysis by Churchill [24] and Pett et al. [26]. Items were developed, validated, and placed in a questionnaire which was tested on 1,048 patients that had undergone surgical treatment. 

Measuring patient participation in treatment is important to improve health services being provided, the dialogue between patients, and healthcare professionals and to identify what areas that could strengthen patient participation. The empirical testing of the instrument reveals a reasonable level of construct validity and reliability and suggests that patient participation is a multidimensional construct that can be measured by 16 items and the following four factors: information dissemination, formulation of options, integration of information, and control. The first factor, information dissemination, explains how information is given and what information the patients receive prior to their operations. The four items relate to general information on the surgical procedure prior to the operation, tests and examinations patients underwent, and benefits and ill effects from surgical treatment [34–36]. The second factor is the formulation of options, in which healthcare professionals explain patients' options, predict the outcome of treatments, and generate lists of possible treatment alternatives. The integration of this information is evident in the third factor. Integration of information, considers patients' opportunities to speak to the physician and nurse before surgery, their opportunities to convey their needs as patients undergoing surgical treatment, and that their needs in connection with the surgical procedure were taken in consideration [13–15]. The last factor is control, which refers to whether the surgical treatment is decided by the healthcare professionals, the patients, or both parties.

The four dimensions had acceptable convergent and discriminant validity, and Cronbach's alphas (ranging from .65 to .80) were more than adequate for this stage of scale development [26]. The four dimensions were moderately correlated indicating further discriminant validity. Compelling evidence was also found for nomological validity, the four dimensions of patient participation were, as expected, significantly correlated to “I had no influence on surgical treatment” (negative correlations) [31] and the UCL coping scale [32]. 

To evaluate the four factors for clinical practice, mean scores for each factor and a total score for the whole instrument have been calculated. The mean scores obtained for the factors information dissemination and integration of information were 5.4 and 5.3, respectively (scale range: 1–7). Patients had opportunities to speak to their responsible physicians and nurses after admission, which supported the patients regarding treatment and made the information understandable.

The factor formulation of options explored to what extent initiatives regarding surgical treatment could be developed in cooperation with patients. The mean score of 2.6 for this factor, which was the lowest among the four factors, showed that involving patients in the formulation of options is given lower emphasis. The last component, control, had a mean score of 3.6. Thus, initiatives in connection with surgical treatment were negotiated to varying degrees with patient cooperation, and patients were not encouraged to participate in decisions regarding surgical treatment (this item had the lowest mean score, 2.9, for the component). Few patients took initiatives to actively participate in decisions regarding treatment. 

In this paper we showed that patient participation in decision making in surgical treatment could be measured using a 16-item measurement instrument. The data were gathered from the patients' perspective. Analyzing from the healthcare professionals' perspective may yield different results and seems suitable for future research. The purpose of the project is to increase the level of knowledge of patient participation in decision making and to identify to which degree patient participation in regard to surgical treatment exists. This knowledge is pivotal in order to further develop the user perspective for surgical patients and to ensure daily routines that consider the patients wishes to participate in the decision making in a plan of treatment.

With our sample size of 1,048 patients, we more than satisfy the recommendation of having at least 10–15 subjects per item to determine a stable factor structure [26]. The study sample was drawn from Norway. The results can only be generalized if the analysis of different samples reveals the same factor structure. Norway is a rather homogeneous country, but some of the characteristics of the Norwegian population might also be found in other countries with a western life style [37] so it might be possible to generalize the results to other areas similar cultures.

The instrument was intended for patients who have undergone surgical treatment, and it is also, in other studies, adapted to healthcare professionals. The instrument is also easy to administer. However, a response rate of 26% and the external validity problems related to the use of a single sample to modify a measure are potential biases related to external validity. Future research may validate the instrument with a new and independent sample. Patient participation in decision making can be difficult to achieve because of incongruence between patient and provider perceptions, knowledge, attitudes, and beliefs [38]. Contrasting the patients' assessments of patient participation with the perspective of the healthcare professionals' assessments can control for the bias of reporting more positive attitudes about patient participation than what can be justified by reality.

## 5. Conclusion

The low degrees of patient participation and high level of paternalism experienced by patients may be interpreted as a need for facilitating patient participation in surgical treatment. This may be more important and beneficial for patients, which may also prove to be economically beneficial for society. The instrument is applicable to patients in surgical wards and is designed to be short and easy to administer. The further establishment of construct validity calls for numerous studies and different approaches over time to generate evidence of the relationships between measures.

## Figures and Tables

**Table 1:** Participants characteristics.

Profession	Ward/surgery	Practice (years)	Earlier surgery	Sex	Age	Education/profession
Physician	Internship	1		F	28	
Physician	Internship	1		M	28	
Physician	Tub/thorax	10		M	40	
Physician	Orthopaedic	27		M	59	
Nurse	Urologic	3		F	27	
Nurse	Gastrologic	2		F	26	
Nurse	Central-surgery unit	21		F	43	
Nurse	Central-surgery unit	28		F	51	
Nurse	Day-surgery unit	32		F	54	
Nurse	Orthopaedic	35		F	58	
Nurse	Day-surgery unit	40		F	62	
Patient	Back operation		Yes, back operation	F	51	BSc nursing
Patient	Ventricle-bowel operation		Yes, two on same issue	F	60	
Patient	Hip replacement		No	F	63	
Patient	Ovaries hysterectomy		Mastectomy cancer mamma	F	65	MSc in teaching
Patient	Kidney transplantation		Yes, kidney transplantation	M	49	Bookkeeper
Patient	Gastrectomy cancer ventriculi		Arm surgery when young	M	61	Engineer
Patient	Urological operation		Yes, kidney stone operation	M	62	MSc engineering

**Table 2:** Demographic characteristics of study sample of patients.

Gender	*N*	%
Men	440	42
Women	603	57.5
Missing	5	.5
Age (years)		
18-19	22	2.1
20–29	69	6.6
30–39	129	12.3
40–49	195	18.6
50–59	225	21.5
60–69	237	22.6
70-	171	16.3
Educational level		
Primary and secondary school (1–9 years)	204	19.5
High school (10–12 years)	445	42.5
College or university (13–16 years)	266	25.4
University (17 years-)	121	11.5
Missing	12	.1
Treatment^2^		
Small operation	373	35.6
Medium operation	427	40.7
Comprehensive/extensive operation	247	23.6
Missing	1	.1
Treatment		
Cancer	153	14.6
Not cancer	892	85.1
Missing	3	.3
Treatment		
General/Endocrine	63	6.0
Orthopaedic	301	28.7
Gynecological	147	14.0
Urological	80	7.6
Gastroenterological	104	9.9
Vein/artery surgery and thoracic surgery	44	4.2
Plastic and hand	127	12.1
Ear, nose, throat	67	6.4
Neuro	32	3.1
Eye	13	1.2
Other	66	6.3
Missing	4	.4
Treatment		
Outpatient (day surgery)	358	34.2
Inpatient (overnight hospital stay)	689	65.7
Missing	1	.1

^2^Small operation means that it lasts less than 30 minutes, medium operation means duration between 30 minutes and two hours, and comprehensive/extensive operation means duration above two hours.

**Table 3:** Distribution of items.

Factors and items	*M *	SD	Skewness	Kurtosis
(1) Information dissemination				
(1) I received written information on the surgical procedure prior to the operation	4.7	2.3	−.61	−1.30
(2) I received oral information on the surgical procedure prior to the operation	5.8	1.5	−.19	3.01
(3) I received general information on the surgical procedure prior to the operation	5.6	1.5	−.15	1.80
(4) I received information on tests and examinations I would undergo during the hospital stay	5.2	1.7	−1.08	.21
(5) I received information on the consequences that surgical treatment could imply	4.7	1.9	−.59	−.94
(6) I received information on the consequences that I could expect when returning home	4.6	2.0	−.56	− 1.03
(7) I received information regarding surgical treatment from a nurse during the hospital stay	4.6	2.0	−.59	−.98
(8) I received information regarding surgical treatment from a physician during the hospital stay	5.3	1.7	−1.27	−.53
(2) Formulation of options				
(9) I had the opportunity to choose the timing of the surgical treatment	2.9	1.9	.74	−.63
(10) I was given several options in connection with the surgical treatment	2.7	1.9	.84	−.72
(11) I was given the opportunity to choose my surgeon	2.1	1.5	1.63	1.86
(12) I was given the opportunity to choose anesthesia	2.4	1.8	1.14	.08
(3) Integration of information				
(13) I had the opportunity to convey my needs as a patient in connection with surgical treatment	4.9	1.7	−.77	−.34
(14) My needs as a patient in connection with the surgical procedure were taken into consideration	4.9	1.7	−.76	−.30
(15) Enough time was spent on information regarding the surgical procedure	5.2	1.6	−1.02	.19
(16) The doctors' answers to my questions were clear and understandable	5.6	1.4	−1.53	2.24
(17) The nurses' answers to my questions were clear and understandable	5.4	1.5	−1.31	1.09
(4) Control				
(18) Initiatives in connection with surgical treatment were worked out with my cooperation	4.0	2.0	−.11	−1.30
(19) I was encouraged to participate in decisions regarding surgical treatment	2.9	2.1	.63	−1.07
(20) I took the initiative to actively participate in decisions regarding treatment	3.7	2.1	.04	−1.46

**Table 4:** Initial eigenvalues, percent of variance and cumulative percent, and the total variance for 20 items and 4 factors.

Factors	Initial eigenvalues		
	Total	% of variance	Cumulative %
(1) Information dissemination	6.300	31.502	31.502
(2) Formulation of options	2.008	10.041	41.543
(3) Integration of information	1.262	6.311	47.854
(4) Control	1.171	5.855	53.709

**Table 5:** Pattern and structure matrix for PAF with an oblimin rotation of the four-factor solution of patient participation in decision making in surgical treatment items. *N* = 1.022.

Factors and items				Factors				
	*(1)*	*(2)*	*(3)*	*(4)*	*(1)*	*(2)*	*(3)*	*(4) *
	Pattern matrix				Structure matrix			
*(1) Information dissemination: *								
Mean 5.4, SD 1.3.								
(1) I received general information on the surgical procedure prior to the operation	**.86**	.01	.04	.04	**.82**	.14	−.42	−.19
(2) I received oral information on the surgical procedure prior to the operation	**.64**	−.03	−.17	−.03	**.63**	.14	−.34	−.15
(3) I received information on tests and examinations I would undergo during the hospital stay	**.64**	.03	.00	.05	**.74**	.17	−.52	−.27
(4) I received information on the consequences that surgical treatment could imply	**.54**	−.05	−.11	.23	**.66**	.19	−.47	−.40
(5) I received written information on the surgical procedure prior to the operation	**.33**	.06	.00	−.14	**.38**	.19	−.25	−.26
*(2) Formulation of options: *								
Mean 2.6, SD 1.3.								
(6) I was given the opportunity to choose my surgeon	.04	**.82**	.04	.10	.16	**.78**	−.19	−.24
(7) I was given the opportunity to choose anesthesia	−.05	**.55**	.01	−.03	.07	**54**	−.14	−.24
(8) I had the opportunity to choose the timing of the surgical treatment	.00	**.49**	−.07	−.08	.17	**55**	−.25	−.32
(9) I was given several options in connection with the surgical treatment	.02	**.25**	−.19	−.31	.26	**.44**	−.39	−.49
*(3) Integration of information:*								
Mean 5.3, SD 1.3.								
(10) My needs as a patient in connection with the surgical procedure were taken into consideration	−.14	.01	**.85**	−.14	.37	30	**−** **.83**	−.41
(11) I had the opportunity to convey my needs as a patient in connection with surgical treatment	−.03	.00	**−** **.70**	−.20	.42	.30	**−** **.76**	−.45
(12) The doctors' answers to my questions were clear and understandable	.22	.02	**−** **.57**	.09	.51	.20	**−** **.67**	−.19
(13) The nurses' answers to my questions were clear and understandable	.13	.04	**−** **.56**	.17	.40	.16	**−** **.58**	−.09
*(4) Control: *							
Mean 3.6, SD 1.6.							
(14) I took the initiative to actively participate in decisions regarding treatment	−.03	.03	.00	**−** **.61**	.16	.28	−.22	**−** **.61**
(15) I was encouraged to participate in decisions regarding surgical treatment	.15	.12	.00	**−** **.60**	.34	.40	−.33	**−** **.69**
(16) Initiatives in connection with surgical treatment were worked out with my cooperation	.23	.00	−.14	**−** **.48**	45	.29	−.44	**−** **.60**
Eigenvalue	5.14	1.87	1.23	1.16				
Cronbach's alpha	.76	.65	.80	.68				

**Table 6:** Pearson correlations among the four factors and related variables.

Factors	(1) Information dissemination	(2) Formulation of options	(3) Integration of information	(4) Control
(1) Information dissemination	—			
(2) Formulation of options	.24	—		
(3) Integration of information	.53	.35	—	
(4) Control	.40	.44	.42	—
UCL coping scale	.53	.29	.66	.39
“I had no influence on treatment”	−.10	−.18	−.16	−.21

All correlations are significant at the .001 level.
